# Impact of nitrogen fertilizer concentrations on growth, yield, and nutrient use efficiency of wolfberry (*Lycium barbarum* L.) in Northwestern China

**DOI:** 10.3389/fpls.2026.1787344

**Published:** 2026-03-04

**Authors:** Mengfei Yuan, Ligang Xu, Jiaxuan Dou, Ying Tang, Xue Tan, Wangbo Xu

**Affiliations:** 1 School of Civil and Hydraulic Engineering, Ningxia University, Yinchuan, Ningxia, China; 2 Key Laboratory for Digital Water Governance of Yellow River Interconnection, Ningxia University, Yinchuan, Ningxia, China; 3 Ningxia Water Conservancy Research Institute, Yinchuan, Ningxia, China; 4 Ningxia Engineering and Technology Research Center for Water-saving and Efficient Dry-crop Agriculture, Yinchuan, Ningxia, China

**Keywords:** drip fertigation, entropy weight-TOPSIS, nitrogen concentration, nutrient use efficiency, soilless cultivation, wolfberry

## Abstract

Nitrogen (N) management is critical for improving productivity and nutrient-use efficiency in substrate-based soilless wolfberry cultivation; therefore, this study aimed to quantify the effects of nutrient-solution N concentration on vegetative growth, nutrient uptake, fruit yield, fruit quality, and nutrient-use efficiency, and to identify an optimal N level for fertigation management. A controlled two-year experiment (2023-2024) was conducted in arid northwestern China with four N concentrations (250, 300, 350, and 400 mg L^-1^) applied via drip fertigation, with three replicates per treatment. Moderate N supply (350 mg L^-1^, T3) enhanced vegetative growth and nutrient uptake and produced the highest yield (2759.65 kg ha^-1^ in 2023 and 2930.93 kg ha^-1^ in 2024), while also improving 100-berry weight and quality-related traits, including β-carotene, crude protein, and essential amino acids. In contrast, the highest N level (400 mg L^-1^, T4) did not further increase yield and was associated with lower nutrient-use efficiency; NUE, PUE, and KUE were higher under low-to-moderate N inputs and declined under high N. An entropy weight-TOPSIS evaluation further ranked T3 as the best overall treatment when multiple indicators were jointly considered, suggesting that optimizing nutrient-solution N concentration to around 350 mg L^-1^ can improve yield and fruit quality while maintaining nutrient-use efficiency under the tested soilless cultivation conditions.

## 1 Introduction

Wolfberry (*Lycium barbarum* L.), a traditional medicinal and economic crop native to northwestern China (Gao et al., 2017; Lu et al., 2021), has received increasing global attention due to its unique nutritional properties (Żołnierczyk et al., 2023) and adaptability to arid and semi-arid environments (Luo et al., 2017). As a core component of desert agriculture, wolfberry production contributes significantly to regional economic development, ecological restoration (Zhang et al., 2023.), and the diversification of functional food products. With the rapid development of sustainable agricultural technologies, soilless cultivation systems are being widely adopted for high-value crops, offering a promising alternative to conventional soil-based production in water-scarce areas.

Soilless cultivation, often coupled with drip fertigation, allows precise control of water and nutrient supply, thereby enhancing crop productivity and resource use efficiency (Savvas, 2002). However, under such controlled conditions, crop performance becomes highly sensitive to the concentrations of nutrients, particularly nitrogen (N), which is a key driver for vegetative growth, photosynthesis, and fruit development (Raj et al., 2021; Sistla and Schimel, 2012). Therefore, optimizing N application under soilless systems is critical to improve yield and quality (Chung et al., 2010; Liang et al., 1997) and also to reduce nutrient leaching, lower environmental risk, and achieve sustainable production goals. Despite the growing application of soilless cultivation in commercial wolfberry production, the mechanisms by which N availability affects plant growth dynamics, nutrient uptake, and quality formation remain inadequately understood.

As a fundamental component of amino acids, nucleic acids, and chlorophyll, N plays a vital role in plant metabolism and structure (Wei et al., 2022; Anas et al., 2020). Adequate N supply promotes leaf area expansion, increases photosynthetic capacity, and enhances carbohydrate translocation to reproductive organs, all of which are essential for yield formation (Zhang et al., 2010, 2020). However, excessive N input under soilless systems can lead to nutrient imbalances, osmotic stress, and reduced fruit quality due to disproportionate vegetative growth. Furthermore, high N levels may induce nitrate accumulation in plant tissues (Quan et al., 2016) and increase nutrient loss by leaching, thus contradicting the principles of efficient and sustainable agriculture. Therefore, the identification of optimal N concentrations which support both growth and resource efficiency is of particular importance in soilless wolfberry cultivation.

Previous studies have reported the effects of N application on various crops under field and greenhouse conditions. For example, research on tomatoes (Cheng et al., 2021; Li et al., 2021) and cucumbers (Wang A., et al., 2019) under nutrient film technique and substrate culture has shown that moderate N levels improve fruit yield and N use efficiency (NUE), while excessive N reduces sugar content and market quality. In wolfberry, field-based studies have suggested that N application above a threshold level does not significantly enhance yield (Lu et al., 2022), and may negatively impact the content of active ingredients, such as betaine and polysaccharides (Chung et al., 2010; Kahsay, 2019). However, such studies have primarily been conducted under traditional soil conditions, and the findings may not be directly transferable to closed, recirculating soilless systems where nutrient dynamics and root zone conditions differ substantially. Moreover, most published research has focused on single response indicators such as yield or nutrient uptake, neglecting the trade-offs and interactions among multiple traits that jointly determine agronomic and economic performance (Zhou et al., 2021; Tong et al., 2024; Li et al., 2025). In particular, few studies have simultaneously evaluated plant growth traits, fruit yield, quality attributes, and nutrient use efficiencies under varying N regimes in soilless wolfberry systems. Additionally, there is a limited integration of multi-criteria decision-making (MCDM) approaches, such as the entropy weight-TOPSIS method (Hao et al., 2022), to assess the comprehensive performance of different N treatments in a robust and objective manner.

To address these knowledge gaps, this study systematically investigated the effects of different N concentrations on growth characteristics, nutrient absorption, fruit yield and quality, and N, phosphorus (P), and potassium (K) use efficiencies in wolfberry under substrate-based soilless cultivation. Field experiments were conducted in a controlled environment located in the Ningxia Hui Autonomous Region of China. Four N concentration levels were applied through drip fertigation, and measurements were taken throughout two consecutive growing seasons.

## 2 Materials and methods

### 2.1 Site description

The experiment was carried out during 2023–2024 at a test site in Ningxia Central Station of China Irrigation Experiment (106°10′E, 38°45′N, 1116 m altitude), Yinchuan City, Ningxia Province, China. The test area has an arid and semi-arid temperate zone climate. Average annual precipitation and evaporation are 198 mm and 1580 mm, respectively. Precipitation is mainly concentrated from July to September (accounting for 60% of the annual precipitation). Average annual temperature and average annual sunshine are 8.5 °C and 2736 h, respectively. The experiment was conducted in the station’s substrate-based soilless cultivation system described in Section 2.3.

### 2.2 Experimental design

The field experiment was designed as a completely randomized design. During the entire growth period, a complete nutrient solution was prepared using standard Hoagland formulation (Fertilizer A and Fertilizer B). N concentration was used as the control variable during nutrient solution preparation, with four treatment levels established: 250 mg L^-1^ (T1), 300 mg L^-1^ (T2), 350 mg L^-1^ (T3), and 400 mg L^-1^ (T4). The ratio schemes of fertilizer A and fertilizer B in different treatments are provided in **Table 1**. Each treatment included three replicates. All other nutrients were kept consistent across treatments, and crop management practices were identical to minimize confounding effects.

**Table 1:** Fertilizer A and fertilizer B application rates used to prepare 1 L nutrient solution at different nitrogen concentrations.

Treatment	Nitrogen concentration (mg L^-1^)	Fertilizer A (mg)	Fertilizer B (mg)	Water (L)
T1	250	1953.28	10.35	1
T2	300	2342.77	12.45	1
T3	350	2730.73	14.55	1
T4	400	3121.77	16.65	1

### 2.3 Soilless cultivation system and drip fertigation management

The experiment was conducted in a purpose-built substrate-based soilless cultivation system at the station, designed for quantifying nutrient demand and operating a closed-loop nutrient-solution recirculation. The facility included two planting troughs and a basement utility room housing independent nutrient-solution supply units. Each planting trough contained cultivation buckets filled with washed perlite as the growth substrate. The troughs measured 16 m × 0.7 m × 0.7 m (L × W × H), and the stainless-steel buckets were 60 cm in diameter and 60 cm in height. The baseline nutrient contents of the perlite were low (total N: 8.42 mg L^-1^; total P: 2.75 mg L^-1^; total K: 0.82 mg L^-1^).

A basement room adjacent to the troughs accommodated 12 independent nutrient-solution supply and recirculation units, each equipped with a low-power submersible pump. Each unit supplied two cultivation buckets. Drainage outlets at the bottom of each bucket were connected to return pipes so that unused nutrient solution flowed back to the corresponding storage tank, forming a closed recirculating loop for each unit.

The wolfberry (*Lycium barbarum* L.) variety in this study was Ningqi-7, four years of age. Drip fertigation was applied using pressure-compensating emitters. Each bucket was fitted with one rectangular and one circular drip ring. Emitters had a flow rate of 4 L h^-1^, with 12 cm spacing between emitters. Irrigation was automatically controlled by a timer, operating every 2 h from 08:00 to 18:00, with each event lasting 10 min. To minimize evaporation and prevent external water inputs, each bucket was sealed with plastic film and secured with bricks; the surface of the troughs was covered with reflective aluminum foil and a black shading net. No other water sources were allowed to enter the system to ensure accurate control of water and nutrient supply.

### 2.4 Measurements

#### 2.4.1 Plant parameters

From bud break to leaf fall, vegetative growth was monitored at 15-day intervals on three representative plants per treatment. Plant height was measured from the substrate surface to the highest point of the canopy using a steel tape. Basal stem diameter was determined with a vernier caliper at a fixed position 5 cm above the substrate surface. For new branches, branch length (from the branch base to the apical tip) and branch diameter (measured at the mid-point of the branch) were recorded. Canopy size was assessed by measuring crown diameter in the east-west and north-south directions; the mean of the two measurements was used as the crown diameter for subsequent analyses. All measurements were conducted using a standardized protocol across treatments to minimize observer-related variability.

#### 2.4.2 Yield

Yields of summer and autumn fruits from representative wolfberry plants were recorded, and the total yield was calculated and expressed as the average yield per hectare. During the summer harvest, ripe fruits were picked in batches as they reached maturity. After harvest, the fruits were processed and dried, and the following parameters were measured: 100-berry dry weight and number of berries per 50 g. The same procedures were applied to the autumn harvest. The 100-berry weight was defined as the mass (g) of 100 randomly selected dried berries. Number of berries per 50 g was evaluated by counting the number of dried berries contained in a 50 g sample, which reflects the commercial classification based on fruit size.

#### 2.4.3 Laboratory analysis

##### 2.4.3.1 Nutrient solution element analysis

To monitor the changes in nutrient composition, solution samples were collected from each of the 12 nutrient-solution tanks every 10 days from early April (bud break) to late October (dormancy stage). Three 550 mL samples were taken from each bucket during each sampling event. After sampling, the remaining solution was discarded and the buckets were thoroughly cleaned. Fresh nutrient solutions were then prepared according to the corresponding treatment formulas and added back to the buckets.

The concentrations of different elements in the nutrient solution were determined using standard analytical techniques. Total N was measured by alkaline potassium persulfate digestion (De Borba et al., 2014) followed by ultraviolet spectrophotometry (Hirt, 1958). Total P was determined by the molybdenum blue colorimetric method (Rodriguez et al., 1994). K content was analyzed using flame photometry (Banerjee and Prasad, 2020). Ca and Mg concentrations were measured using EDTA complexometric titration (Kimaru et al., 2018).

##### 2.4.3.2 Quality

The contents of betaine, β-carotene, protein, and essential amino acids in dried wolfberry fruits were determined using standard analytical methods. Betaine was measured using high-performance liquid chromatography (HPLC) equipped with a C18 reversed-phase column and UV detection. β-Carotene content was quantified by ultraviolet-visible (UV-Vis) spectrophotometry based on its absorbance at 450 nm. Crude protein content was determined using the Kjeldahl method, with the total N content converted to protein by applying a conversion factor of 6.25.

Essential amino acids (EAAs) were measured by acid hydrolysis followed by HPLC analysis with pre-column derivatization using o-phthalaldehyde. The quantified EAAs included lysine (Lys), histidine (His), phenylalanine (Phe), methionine (Met), threonine (Thr), isoleucine (Ile), leucine (Leu), and valine (Val). The total EAA content was expressed as the sum of these eight amino acids on a dry weight basis (g 100 g^-1^).

#### 2.4.4 NUE, PUE and KUE

NUE, PUE, and KUE were determined using the following Equations 1–3:

(1) NUE=Y/NU

(2) PUE=Y/PU

(3) KUE=Y/KU

Where NUE is nitrogen use efficiency (kg kg ^-1^); PUE is phosphorus use efficiency (kg kg ^-1^); KUE is potassium use efficiency (kg kg ^-1^); Y is the yield (t ha ^-1^); NU is crop nitrogen uptake (kg ha ^-1^); PU is crop phosphorus uptake (kg ha ^-1^); KU is crop potassium uptake (kg ha ^-1^).

### 2.5 Comprehensive evaluation methods

In order to comprehensively evaluate the effects of different treatments on crop growth and yield, with yield, 100 grain weight, number of berries per 50 g and element absorption (N, P, K) as evaluation indexes, Entropy Weight-TOPSIS (He et al., 2024; Li et al., 2023) was used to comprehensively evaluate different treatments. The detailed equations for entropy weight determination are as follows (Equations 4–7):

(4) $$W_{m}=\frac{1-E_{m}}{s-\sum E_{m}}$$

(5) $$E_{m}=-\ln{\left(t\right)}^{-1}\sum_{n=1}^{t}p_{mn}ln\left(p_{mn}\right)$$

(6) $$p_{mn}=\frac{y_{mn}}{\sum_{n=1}^{t}y_{mn}}$$

(7) $$y_{mn}=\frac{r_{mn}-\min(r_{m})}{\max(r_{m})-\min(r_{m})}(m=1,\;2\ldots ,s)$$

where, *W_m_* represents the entropy weight; *E_m_* represents the entropy value for the *m^th^*; *P_mn_* represents the proportion of the *n^th^* evaluation object under the *m^th^* index; *y_mn_* represents the dimensionless value of the *i^th^* evaluation object under *j^th^* index; *r_mn_* represents the value of *n^th^* evaluation object of the *m^th^* index.

The relative proximity C was used as the score index of each evaluation scheme. The detailed equations for deciding the relative proximity are as follows (Equations 8–11):

(8) $$C=\frac{D^{-}}{D^{+}+D^{-}}$$

(9) $$D^{-}=\sqrt{{\sum_{m=1}^{s}\left(z_{mn}-z_{m}^{-}\right)}^{2}}\;(n=1,\;2\ldots ,t)$$

(10) $$D^{-}=\sqrt{{\sum_{m=1}^{s}\left(z_{mn}-z_{m}^{+}\right)}^{2}}\;(n=1,\;2\ldots ,t)$$

(11) $$z_{mn}=\frac{r_{mn}}{\sqrt{\sum_{n=1}^{t}r_{mn}^{2}}}\cdot W_{m}\;(m=1,2\ldots ,s;n=1,2\ldots ,t)$$

### 2.6 Statistical analyses

Data are presented as mean ± standard error (SE) with three replicates. Treatment effects were tested using one-way analysis of variance (ANOVA) separately for each year. When significant differences were detected, means were compared using the least significant difference (LSD) test at P< 0.05. All analyses were conducted using appropriate statistical software.

## 3 Results

### 3.1 Growth indexes

**Figure 1:**
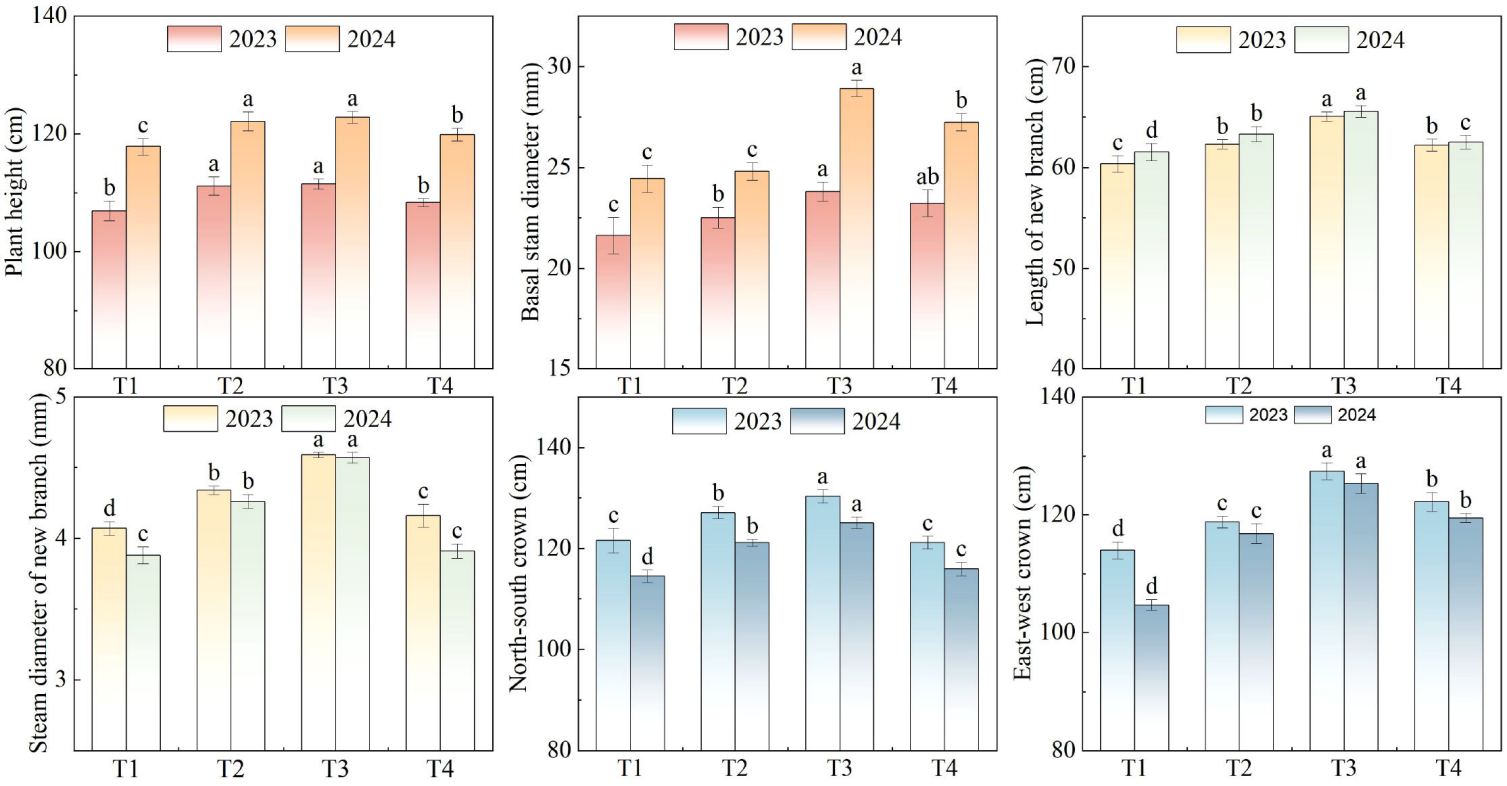
Effects of nitrogen (N) concentration in nutrient solution on wolfberry vegetative growth traits in 2023 and 2024. Shown are plant height, basal stem diameter, length of new branch, diameter of new branch, and canopy crown (east-west and north-south) under four N treatments (T1-T4: 250, 300, 350, and 400 mg L^-1^). Values are means ± SD (n = 3). Different lowercase letters indicate significant differences among treatments within the same year (LSD test, p< 0.05).

Wolfberry growth parameters differed significantly among N treatments (**Figure 1**). Growth parameters initially increased and then decreased with increasing N concentration. The highest plant height value appeared in T3, at 111.47 cm and 122.79 cm in 2023 and 2024, respectively. The minimum values for the two experimental years were recorded in T1, which were 106.90 cm and 117.84 cm, respectively. Plant height under T3 treatment was 4.24% greater compared to T1 treatment. Basal stem diameter also reached its maximum in T3, being 14.16%, 11.15%, and 4.31% higher than T1, T2, and T4, respectively (**Figure 1**).

For the length of new branch, T3 resulted in the greatest values, reaching 65.04 cm in 2023 and 65.56 cm in 2024; the lowest values in both 2023 and 2024 were observed in T1, measuring 60.35 cm and 61.52 cm, respectively (**Figure 1**). Under T3 treatment, the stem diameter of new branch reached the highest value, exceeding those of T1, T2, and T4 by 0.61 mm, 0.28 mm, and 0.55 mm, respectively (**Figure 1**). In terms of north-south canopy width, the treatments ranked in the following order: T3, T4, T2, and T1. T3 showed the greatest east-west canopy, with measurements of 130.34 cm and 125.06 cm in two growing seasons. (**Figure 1**).

### 3.2 Elemental uptake from nutrient solution

#### 3.2.1 Macronutrients

**Figure 2:**
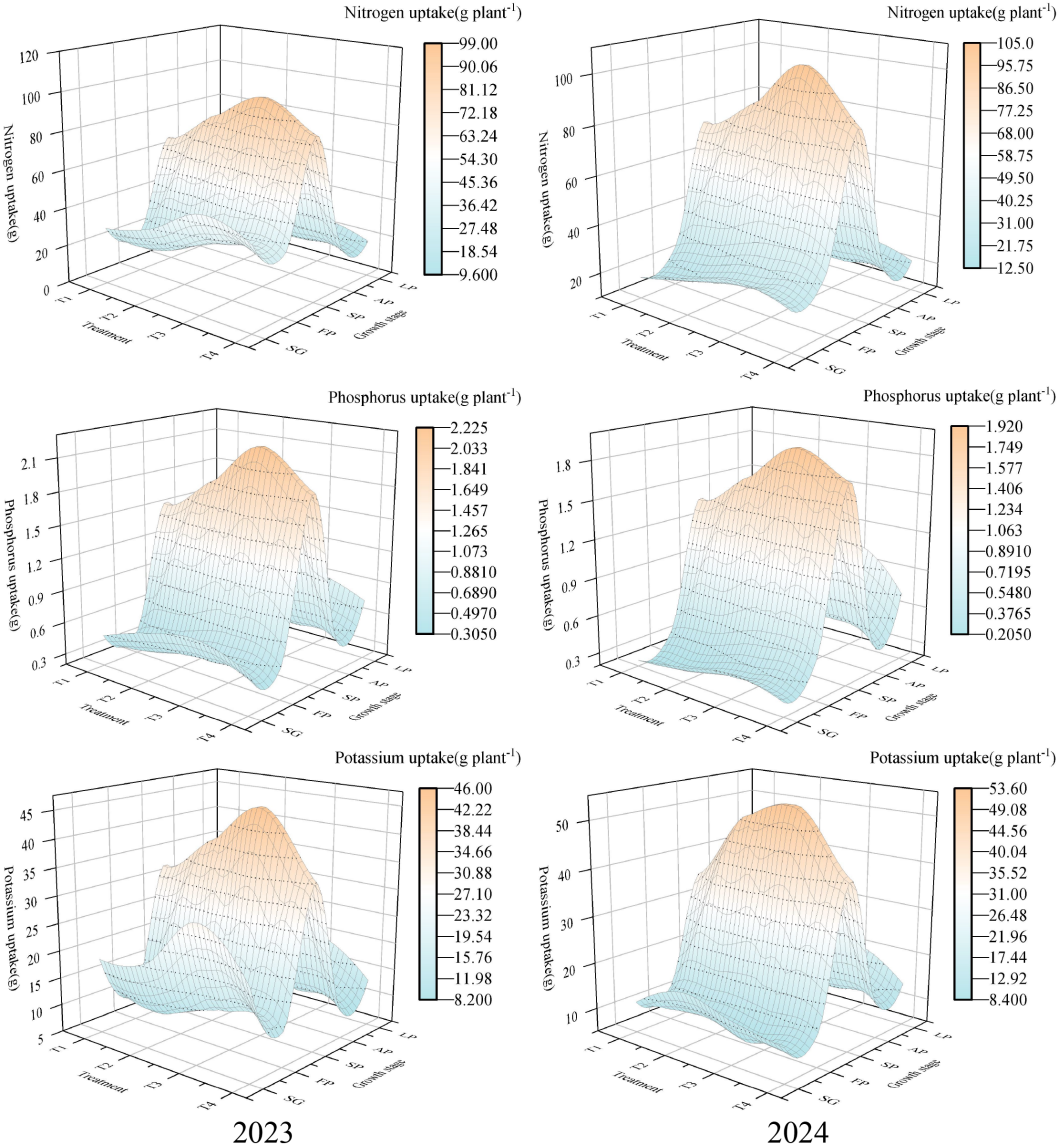
Absorption percentages of macronutrients at different treatment and growth stages. The proportions of total seasonal uptake are shown for N, P, and K across five growth stages (SG, spring shoot growth; FP, flowering period; SP, summer fruit period; AP, autumn fruit period; LP, leaf fall period). Treatments: T1-T4 correspond to 250, 300, 350, and 400 mg L^-1^ N in the nutrient solution.

Total absorption of N, P, K under different treatments showed significant differences (*p*< 0.05, **Table 2**). The highest total N uptake was in T3 (236.25 g plant^-1^ in 2023 and 228.82 g plant^-1^ in 2024), and the lowest total N uptake occurred in T1 (148.52 g plant^-1^ in 2023 and 155.92 g plant^-1^ in 2024) (**Table 2**). Total P uptake was also greatest in T3 (5.10 g plant^-1^ in 2023; 4.50 g plant^-1^ in 2024) (**Table 2**). The order of total K uptake in two growing seasons was T3 > T2 > T4 > T1 (**Table 2**). Uptake of macronutrients increased initially and then decreased with increasing N concentration (**Figure 2**). Stage-wise analysis indicated that macronutrient uptake was concentrated during the summer fruiting period, during which N, P, and K uptake each accounted for more than 38% of the respective seasonal totals (**Figure 2**). This indicates that the application of an appropriate fertilizer concentration during the summer fruiting period enhances nutrient absorption by wolfberry.

**Table 2:** Total elemental uptake across different treatments.

Years	Treatment	Macronutrients (g plants^-1^)			Secondary macronutrients (g plant^-1^)	
		N	P	K	Ca	Mg
2023	T1	148.52 ± 2.09d	3.49 ± 0.28d	85.88 ± 1.13d	89.96 ± 1.36d	29.76 ± 0.52d
	T2	186.61 ± 2.64c	4.49 ± 0.18b	99.38 ± 1.65b	103.85 ± 1.33b	34.10 ± 0.42b
	T3	236.25 ± 4.41a	5.10 ± 0.23a	121.80 ± 1.56a	113.88 ± 1.28a	39.90 ± 0.43a
	T4	199.98 ± 2.39b	4.17 ± 0.35c	90.34 ± 1.37c	94.29 ± 1.21c	33.17 ± 0.78c
2024	T1	155.92 ± 1.06c	3.01 ± 0.13d	88.30 ± 0.49d	96.45 ± 1.28c	41.76 ± 0.58d
	T2	188.73 ± 2.20b	3.54 ± 0.28c	112.48 ± 1.15b	104.31 ± 1.21b	48.38 ± 0.53b
	T3	228.82 ± 2.83a	4.50 ± 0.27a	116.60 ± 1.17a	109.47 ± 1.22a	53.22 ± 0.76a
	T4	190.44 ± 1.05b	3.68 ± 0.18b	90.95 ± 1.23c	96.25 ± 1.40c	44.92 ± 0.79c

Different lowercase letters indicate significant differences among treatments within the same year (LSD test, p < 0.05).

#### 3.2.2 Secondary macronutrients

**Figure 3:**
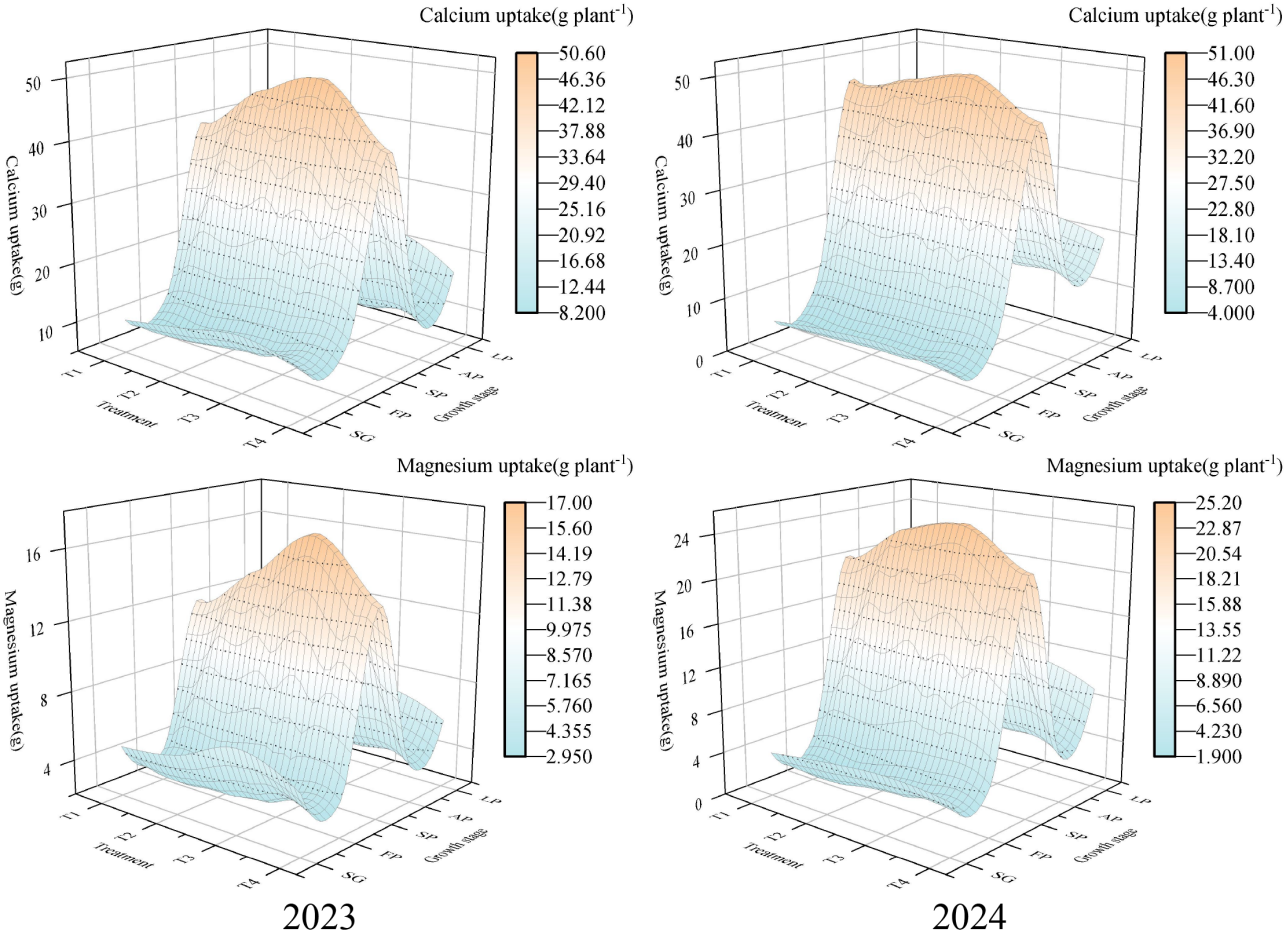
Absorption percentages of secondary macronutrients at different treatment and growth stages. The proportions of total seasonal uptake are shown for Ca and Mg across five growth stages (SG, spring shoot growth; FP, flowering period; SP, summer fruit period; AP, autumn fruit period; LP, leaf fall period). Treatments: T1-T4 correspond to 250, 300, 350, and 400 mg L^-1^ N in the nutrient solution.

The total absorption of Ca, Mg under different treatments showed significant differences (*p*< 0.05, **Table 2**). The highest Ca uptake occurred in T3 (113.88 g plant^-1^ in 2023 and 109.47 g plant^-1^ in 2024); the lowest Ca uptake appeared in T1 (89.96 g plant^-1^) in 2023 and T4 (96.25 g plant^-1^) in 2024. The total Mg uptake in two growing seasons was ranked as follows: T3 > T2 > T4 > T1 (**Table 2**). Ca and Mg uptake was also concentrated during the summer fruiting period. In 2023, Ca and Mg accounted for 44%-46% and 41%-43% of their respective totals, whereas in 2024 the corresponding ranges were 47%-49% for Ca and 48%-50% for Mg (**Figure 3**).

#### 3.2.3 Microelement

**Figure 4:**
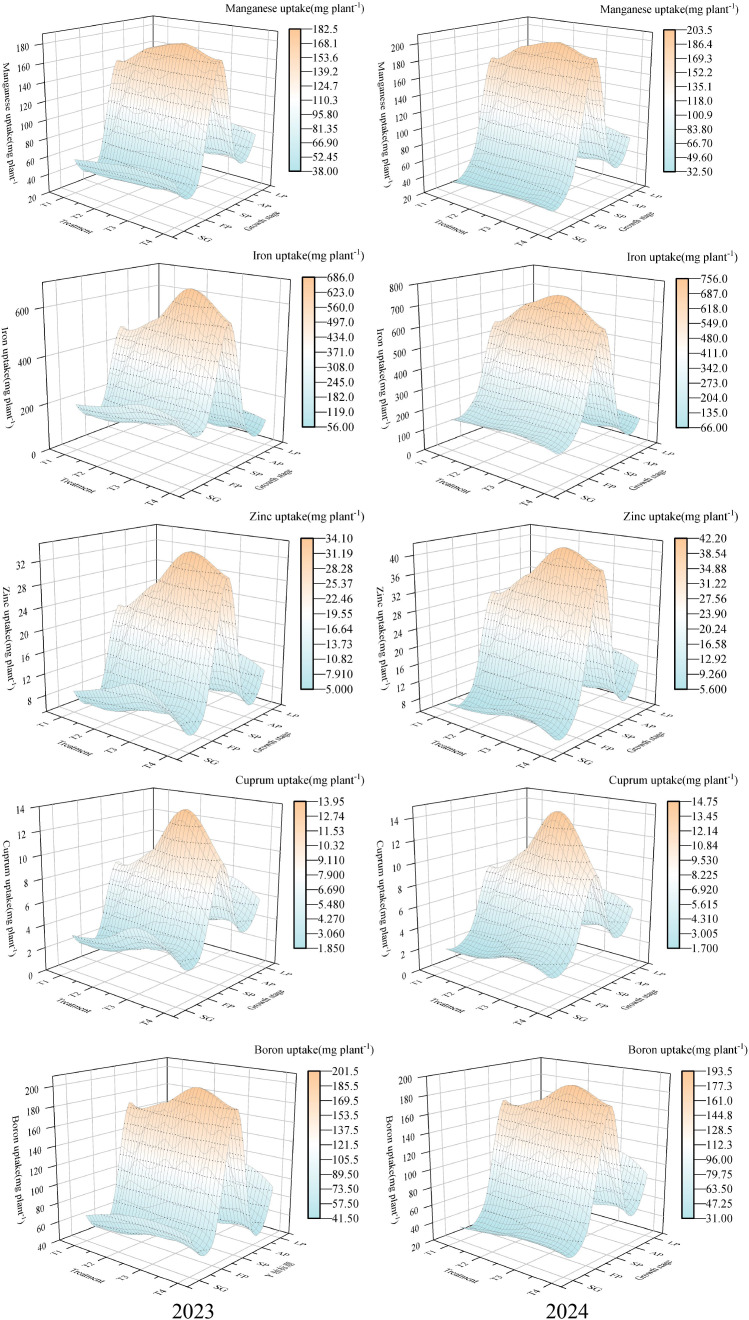
Absorption percentages of micronutrients at different treatment and growth stages. The proportions of total seasonal uptake are shown for Mn, Fe, Zn, Cu, and B across five growth stages (SG, spring shoot growth; FP, flowering period; SP, summer fruit period; AP, autumn fruit period; LP, leaf fall period). Treatments: T1-T4 correspond to 250, 300, 350, and 400 mg L^-1^ N in the nutrient solution.

The effect of different N concentrations on the uptake of microelements showed significant differences (*P*< 0.05, **Table 3**). In both growing seasons, Mn uptake was highest under the T3 treatment, with 450.80 mg plant^-^¹ in 2023 and 469.01 mg plant^-^¹ in 2024. In 2023, the order of Fe uptake was T3 > T4 > T1 > T2, while in 2024, the order was T3 > T2 > T4 > T1. For Zn, uptake in all treatments exceeded 53.07 mg plant^-^¹ in both growing seasons, with the highest uptake observed in T3, reaching 80.84 mg plant^-^¹ in 2023 and 93.30 mg plant^-^¹ in 2024. The lowest uptake occurred in T1, with an average of 59.16 mg plant^-^¹ in both growing seasons. For Cu and B, the uptake in both growing seasons followed the order: T3 > T4 > T2 > T1. As shown in **Figure 4**, the highest proportion of trace element uptake occurred during the summer fruiting stage in both 2023 and 2024, exceeding 38% and 40% respectively.

**Table 3:** The absorption of micronutrients by wolfberry under different nitrogen treatments.

Years	Treatment	Micronutrients (mg plants^-1^)				
		Mn	Fe	Zn	Cu	B
2023	T1	376.61 ± 5.77c	1063.58 ± 33.52d	53.07 ± 1.33d	20.45 ± 0.36d	425.90 ± 4.07d
	T2	420.55 ± 4.20b	1148.60 ± 24.39c	65.23 ± 1.01c	24.90 ± 0.33c	455.99 ± 5.08c
	T3	450.80 ± 4.32a	1431.94 ± 55.58a	80.84 ± 1.12a	35.08 ± 0.46a	525.77 ± 4.13a
	T4	421.46 ± 4.37b	1236.23 ± 39.89b	68.18 ± 0.91b	26.63 ± 0.38b	472.53 ± 3.78b
2024	T1	394.94 ± 5.12c	1260.05 ± 45.35c	65.25 ± 1.36d	19.86 ± 0.29c	374.93 ± 2.97d
	T2	439.24 ± 4.32b	1383.78 ± 41.08c	77.33 ± 1.05c	24.81 ± 0.23b	402.61 ± 4.38c
	T3	469.01 ± 3.31a	1515.01 ± 28.05a	93.30 ± 1.10a	34.04 ± 0.52a	476.59 ± 3.61a
	T4	439.26 ± 4.26b	1284.69 ± 35.17bc	81.37 ± 1.00b	24.83 ± 0.35b	420.25 ± 3.46b

Different lowercase letters indicate significant differences among treatments within the same year (LSD test, p < 0.05).

### 3.3 Yield and quality

#### 3.3.1 Yield, 100-berry weight and number of berries per 50 g

**Figure 5:**
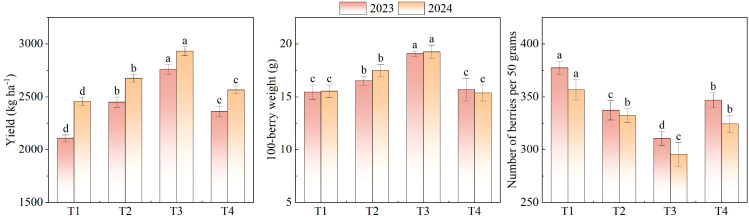
The yield, 100-berriy weight and the number of berries per 50g of wolfberry under different treatments. T1-T4 correspond to 250, 300, 350, and 400 mg L^-1^ N. Values are means ± SD (n = 3). Different lowercase letters indicate significant differences among treatments within the same year (LSD test, p< 0.05).

Significant differences in yield were observed among the different treatments. Wolfberry yield increased initially and then decreased with increasing N concentration, namely T3 > T2 > T4 > T1. The highest yields were recorded under T3 (2759.65 kg ha^-1^ in 2023; 2930.93 kg ha^-1^¹ in 2024) (**Figure 5**), which indicates that an optimal N supply contributes to enhanced wolfberry yield. For 100-berry weight, the treatment performance ranked as T3 > T2 > T4 > T1 in 2023, while in 2024, the ranking shifted to T3 > T2 > T1 > T4, but there was no significant difference between T1 and T4 in 2023 and 2024 (**Figure 5**). The 100-berry weight in T3 was 19.07 g in 2023 and 19.24 g in 2024. The number of berries per 50 g followed the order T1 > T4 > T2 > T3 in 2023, and T1 > T2 > T4 > T3 in 2024 (**Figure 5**).

#### 3.3.2 Quality

**Figure 6:**
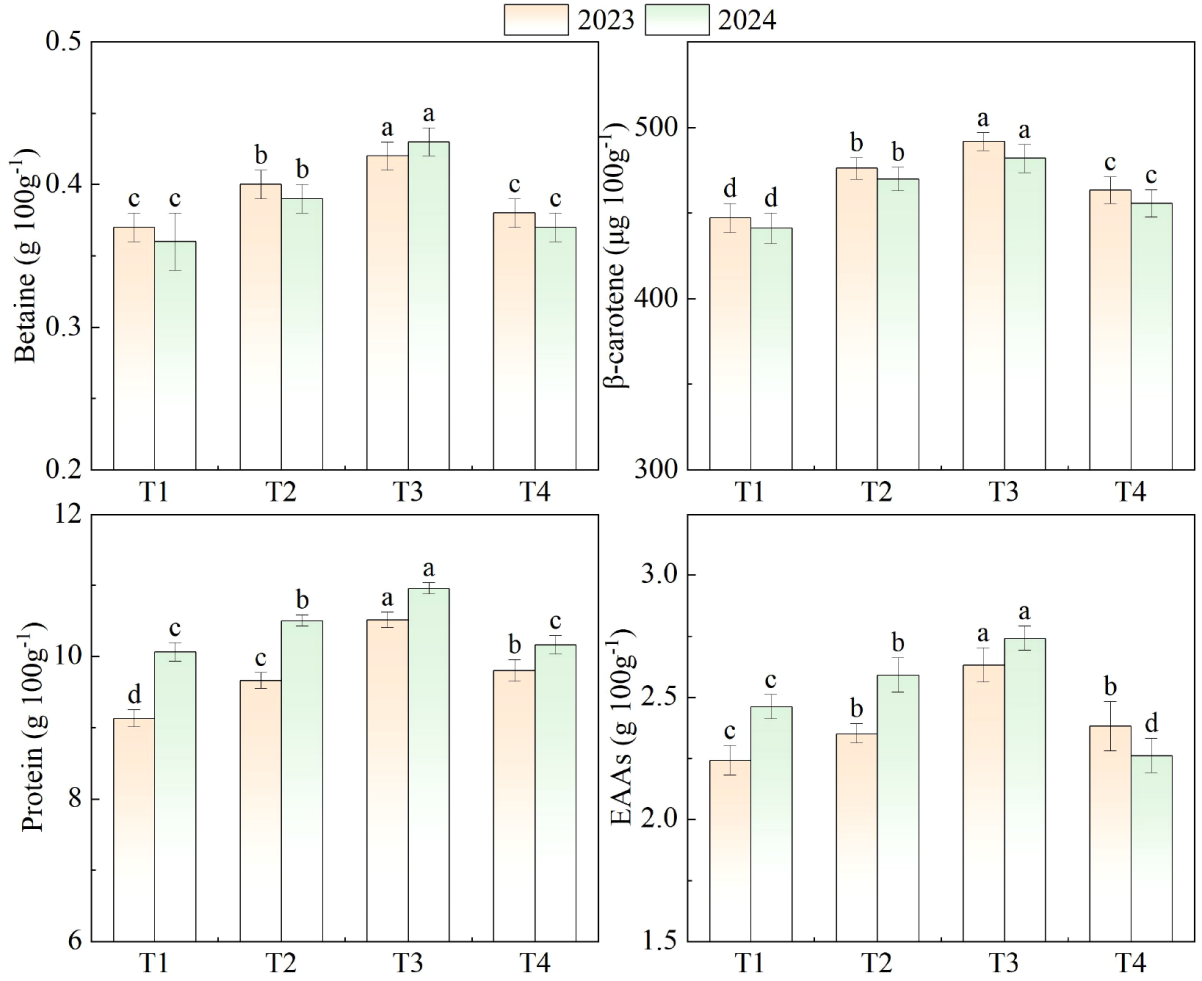
Quality indicators of wolfberry under different treatments. T1-T4 correspond to 250, 300, 350, and 400 mg L^-1^ N. Values are means ± SD (n = 3). Different lowercase letters indicate significant differences among treatments within the same year (LSD test, p< 0.05).

Significant differences in betaine, β-carotene, protein, and total content of EAAs were observed among the different treatments (**Figure 6**). Comparing betaine in different N concentrations, the highest betaine values in two growing seasons were in T3 (average of 0.42 g 100 g^-1^ for two seasons). The lowest betaine values appeared in T1 (average of 0.37 g 100 g^-1^ two seasons). β-carotene consistently followed the order T3 > T2 > T4 > T1 in the two growing seasons. With an increase in N concentration, protein content first increased and then decreased. The highest protein content appeared in T3 (average of 10.73 g 100 g^-1^ for two seasons). In both growing seasons, the T3 treatment resulted in the highest content of EAAs, with values of 2.63 g 100 g^-1^ in 2023 and 2.74 g 100 g^-1^ in 2024. The lowest content was observed in the T1 treatment in 2023 (2.24 g 100 g^-1^) and in T4 in 2024 (2.26 g 100 g^-1^).

### 3.4 Nitrogen use efficiency, phosphorus use efficiency, and potassium use efficiency

There were significant differences (*p*<0.05, **Table 4**) in NUE, PUE, and KUE under different treatments. NUE, PUE, and KUE initially decreased and then increased with increasing N concentration. The highest NUE occurred in treatment T1 in two growing seasons, with value of 4.26 kg kg^-1^ in 2023 and 4.73 kg kg^-1^in 2024. The lowest NUE was observed in T3, with the value of 3.51 kg kg^-1^ in 2023 and 3.91 kg kg^-1^ in 2024. In 2023, the PUE followed the order T1 > T4 > T2 > T3 while, in 2024, the sequence was T1 > T2 > T4 > T3. In both growing seasons, the highest KUE was observed under the T4 treatment, with values of 7.85 kg kg^-1^ in 2023 and 8.47 kg kg^-1^ in 2024 (**Table 4**).

**Table 4:** NUE, PUE and KUE under different treatments.

Years	Treatment	NUE (kg kg^-1^)	PUE (kg kg^-1^)	KUE (kg kg^-1^)
2023	T1	4.26 ± 0.11a	181.50 ± 4.02a	7.37 ± 0.23c
	T2	3.94 ± 0.13b	163.82 ± 2.96c	7.40 ± 0.28b
	T3	3.51 ± 0.09c	162.53 ± 2.82c	6.80 ± 0.11b
	T4	3.55 ± 0.07c	170.31 ± 3.99b	7.85 ± 0.19a
2024	T1	4.73 ± 0.15a	245.50 ± 3.37a	8.35 ± 0.16a
	T2	4.26 ± 0.16b	227.16 ± 2.79b	7.14 ± 0.38c
	T3	3.91 ± 0.08d	198.96 ± 3.98d	7.68 ± 0.19b
	T4	4.05 ± 0.12c	209.19 ± 2.90c	8.47 ± 0.18a

Different lowercase letters indicate significant differences among treatments within the same year (LSD test, p < 0.05).

### 3.5 Comprehensive evaluation of different treatments

The comprehensive yield and resource-use efficiency scores in 2023 and 2024, calculated by using Entropy Weight-TOPSIS, are shown in **Table 5**. Entropy Weight-TOPSIS showed that T3, T1, and T2 ranked first, second and third, respectively, whereas T4 ranked lasted in 2023. Similarly, T3, T2, and T1 were ranked first, second and third, whereas T4 ranked lasted in 2024. T3 showed the highest comprehensive score under soilless cultivation, based on traits such as yield, 100-berry weight, number of berries per 50 g and NUE, PUE, and KUE.

**Table 5:** TOPSIS score and rank of different treatments.

Treatment	2023				2024			
	D^+^ (Distance to positive ideal solution)	D^-^ (Distance to negative ideal solution)	C_i_ (Relative closeness)	Rank	D^+^ (Distance to positive ideal solution)	D^-^ (Distance to negative ideal solution)	C_i_ (Relative closeness)	Rank
T1	0.231	0.201	0.464	2	0.226	0.171	0.43	3
T2	0.206	0.124	0.376	3	0.148	0.157	0.515	2
T3	0.207	0.23	0.526	1	0.165	0.231	0.583	1
T4	0.24	0.109	0.312	4	0.23	0.095	0.293	4

## 4 Discussion

### 4.1 Growth parameters and nutrient uptake

Plant morphological indicators can provide an intuitive reflection of plant growth status (Watanabe et al., 2017; Zheng et al., 2024). The present study showed that plant height, basal stem diameter, length of new branch, stem diameter of new branch, north-south canopy and east-west canopy increased initially with increasing irrigation quota and irrigation frequency, then subsequently decreased, indicating that moderate N concentration optimized wolfberry growth, as reflected by these morphological indicators. The values of growth parameters were the highest under T3 (with a N concentration of 350 mg L^-1^). This treatment led to the highest plant height and stem diameter, and it promoted the growth of new branches (**Figure 1**). This aligns with the results of other crop studies. For instance, Xiao et al. (2023) demonstrated that N concentration significantly influences tomato growth, particularly in terms of plant height and leaf area, with suitable N concentrations enhancing the growth rate. According to Zhu et al. (2016), maize plant height increased with rising N levels within a specific range, while excessive N concentrations suppressed maize growth. This occurred because excessive N fertilization restricted root development and impaired the roots’ ability to absorb water (Feng et al., 2016), ultimately limiting crop growth. N nutrition was closely linked to the level of cytokinins. Insufficient nutrient supply resulted in a decrease in cytokinin content in plant leaves, while N nutrition promoted the accumulation of cytokinins (Samuelson and Larsson, 1993). Therefore, under low N conditions, the reduced cytokinin content inhibited plant growth and development, leading to stunting and wilting.

Elements such as N, P, and K are the most essential nutrients for plant growth and development (Ma et al., 2022; Guo et al., 2019). Macronutrients alone are insufficient to meet the growth requirements of crops; secondary macronutrients are also necessary to meet demand (Grzebisz, Zielewicz, & Przygocka-Cyna 2023; Bekele and Birhan, 2021). This study found that with an increasing N concentration, the uptake of different elements by wolfberry first increased and then decreased. Under T3, the uptake of N, P, K, Ca, and Mg reached their maximum values, indicating that an appropriate N concentration promotes the absorption of various elements by wolfberry. The highest proportion of uptake for both macronutrients and secondary macronutrients occurred during the summer fruiting period. This period is crucial for fruit development in wolfberry and it serves as a key stage for balancing vegetative and reproductive growth. Therefore, meeting the nutrient requirements of wolfberry during the summer fruiting stage is critical to improve both yield and quality.

### 4.2 Yield and quality

N management played a crucial role in regulating wolfberry yield components, including total yield, 100-berry weight, and number of berries per 50 g, as well as determining fruit quality attributes. Across two growing seasons, the results clearly indicated that moderate N application (T3, 350 mg L^-1^) significantly enhanced wolfberry yield, while excessive or insufficient N levels led to suboptimal outcomes. Yield performance under different N treatments followed a typical parabolic response pattern, consistent with findings in tomatoes (Li et al., 2021) under controlled environments. The highest yields recorded in T3 (2759.65 kg ha^-1^ in 2023 and 2930.93 kg ha^-1^ in 2024) reflect the benefits of sufficient N availability for maximizing reproductive growth and dry matter translocation toward fruits. In contrast, the lowest yields observed in T1 (250 mg L^-1^) suggest that N deficiency limits canopy expansion, photosynthetic capacity, and assimilate supply for fruit development, a trend similarly reported by Wang et al. (2019) in cucumber under soilless conditions. In terms of fruit weight, 100-berry weight under T3 was significantly higher than in other treatments, reaching 19.07 g in 2023 and 19.24 g in 2024. A higher 100-berry weight is indicative of larger fruit size and better market classification, which directly influences commercial value. These findings agree with previous studies showing that moderate N improves cell expansion and water accumulation in fruit tissues (Zhang et al., 2023). Notably, although T1 exhibited a higher number of berries per 50 g, suggesting smaller fruit size, it did not translate into better yield performance, underscoring the importance of balancing fruit number and fruit weight for overall productivity.

Regarding fruit quality, N concentration also exerted significant effects on key biochemical attributes. Sweet beet alkaloid (betaine) content showed a declining trend with increasing N levels, possibly due to the dilution effect caused by accelerated vegetative growth under high N supply. This phenomenon was similarly observed in fertilization experiments on other *Lycium* species (Zhou et al., 2021). The highest β-carotene concentration under T3 demonstrates that moderate N promotes carotenoid biosynthesis, which is essential for enhancing fruit nutritional value and antioxidant properties. Protein content and total content of EAAs were also maximized under T3. Adequate N availability likely stimulated protein biosynthesis and amino acid accumulation, improving the nutritional profile of wolfberry fruits. The higher EAA content under moderate N supply aligns with the findings of Xu et al., 2012 in other horticultural crops, where N optimization enhanced both yield and quality traits simultaneously.

### 4.3 Nutrient use efficiency

NUE, PUE, and KUE are key determinants of sustainable agricultural productivity (Zhang et al., 2019), especially under soilless cultivation systems where precise nutrient management is critical. In this study, NUE, PUE, and KUE were significantly influenced by N concentration applied through drip fertigation. Moderate N concentration (350 mg L^-1^, T3) optimized both crop productivity and nutrient efficiency, whereas higher or lower N inputs reduced overall nutrient use efficiencies. High NUE is associated with improved N assimilation and reduced N loss, contributing to both yield improvement and environmental sustainability (Xu et al., 2012). Estimates suggest that a 1% increase in crop NUE could result in an annual saving of approximately 1.1 billion USD (Kant et al., 2011). Excessive N application, as observed under T4 treatment, can lead to luxury consumption, nitrate accumulation, and lower NUE due to imbalance between vegetative and reproductive growth (Zhang et al., 2023). These findings align with earlier studies in greenhouse-grown crops under soilless cultivation systems (Rouphael and Colla, 2005), where moderate N supply enhanced biomass allocation towards economically valuable plant parts, improving yield and nutrient use efficiency. Similarly, PUE exhibited a decreasing trend with increased N concentration beyond the optimum. Efficient P uptake is crucial for root development and energy transfer processes (Vance et al., 2003). Inadequate management may lead to P fixation or leaching even under closed soilless systems, thereby reducing effective PUE. KUE is intimately linked with water regulation and stress tolerance mechanisms (Wang et al., 2013). The observed improvements in KUE under moderate N conditions suggest better coordination between water uptake, osmotic adjustment, and carbohydrate translocation, ultimately supporting fruit development and quality.

### 4.4 Comprehensive evaluation

The comprehensive performance evaluation using the entropy weight-TOPSIS method effectively integrated multiple agronomic and physiological indicators, including yield, 100-berry weight, berry size, and NUE, PUE, and KUE, to assess the overall impact of different N treatments on wolfberry under soilless cultivation. This section is included because these indicators may not change in the same direction among treatments; therefore, a single-trait comparison may be insufficient to identify an overall optimal N level. This approach provided an objective framework to rank treatments by simultaneously considering production output and resource use sustainability. The results showed that the T3 treatment (350 mg L^-1^) consistently ranked highest over two growing seasons, confirming that moderate N supply optimized both yield and nutrient use efficiency. Excessive (T4) or insufficient (T1) N treatments, although advantageous in isolated traits, failed to achieve comparable overall performance. This finding agrees with the principles outlined by Zhou et al. (2006), who emphasized the importance of applying multi-criteria decision analysis to balance competing agricultural objectives. Furthermore, the entropy weighting approach provided an objective method to assign indicator importance based on data variability, minimizing subjective bias in the evaluation process (Zardari et al., 2015).

## 5 Conclusion

This study evaluated how nitrogen (N) concentration in nutrient solution affects vegetative growth, nutrient uptake, yield, fruit quality, and nutrient-use efficiencies of wolfberry under substrate-based soilless cultivation. Across two consecutive growing seasons, the application of 350 mg L^-1^ N (T3) consistently achieved optimal performance. T3 treatment resulted in the highest grain yield, with corresponding improvements in 100-berry weight, reduced berry count per 50 g, and enhanced fruit quality attributes, including elevated contents of β-carotene, crude protein, and essential amino acids. Furthermore, NUE, PUE, and KUE were significantly influenced by N concentration, with moderate N supply promoting better resource utilization. The entropy weight-TOPSIS-based comprehensive evaluation ranked T3 as the most effective treatment, demonstrating a strong balance between productivity, quality, and nutrient use efficiency. The results indicated that moderate N concentration not only promoted vegetative growth but also optimized nutrient absorption during critical reproductive stages. In conclusion, applying 350 mg L^-1^ N through drip fertigation under soilless cultivation effectively matched crop N demand, maximizing both yield and quality. Future work should validate this N strategy across longer production cycles and under different environmental conditions, and examine its interactions with other nutrients and fertigation scheduling in closed-loop systems.

## Data Availability

The raw data supporting the conclusions of this article will be made available by the authors, without undue reservation.
